# Weight Loss History and Its Association with Self-Esteem and Eating Behaviors in Adolescents and Young Adults with Obesity

**DOI:** 10.1159/000529267

**Published:** 2023-01-25

**Authors:** Liisa Tolvanen, Anne Christenson, Helén Eke, Stephanie E. Bonn, Ylva Trolle Lagerros

**Affiliations:** ^a^Division of Clinical Epidemiology, Department of Medicine Solna, Karolinska Institutet, Stockholm, Sweden; ^b^Center for Obesity, Academic Specialist Center, Stockholm Health Services, Stockholm, Sweden

**Keywords:** Eating behavior, Obesity, Self-esteem, Weight loss, Young adult

## Abstract

**Introduction:**

Previous weight loss attempts in young people with obesity may have influenced their beliefs about themselves and contributed to maladaptive eating behaviors. Therefore, we aimed to investigate the association between previous weight loss with self-esteem and different eating behaviors in adolescents and young adults with obesity seeking specialty obesity care.

**Methods:**

We performed a cross-sectional study, where a total of 224 participants with obesity, aged 16–25, self-reported the amount and the frequency of previous weight loss of 5 kg or more. Self-esteem was assessed with Rosenberg's Self-Esteem Scale and eating behavior with the Three-Factor Eating Questionnaire-Revised21. Linear regression was used to analyze associations between the amount of weight loss (no weight loss, 5–10 kg, and >10 kg) and the frequency of weight loss ≥5 kg (0, 1, and ≥2 times) with self-esteem and eating behaviors.

**Results:**

We found that both those who had lost 5–10 kg and those who had lost ≥5 kg twice or more, had statistically significantly higher cognitive restraint eating scores β = 7.03 (95% CI: 0.004–14.05) and β = 8.32 (95% CI: 1.20–15.43), respectively, compared to those who reported no previous weight loss. No other statistically significant associations were found.

**Conclusion:**

Previous weight loss in adolescents and young adults with obesity may be associated with a higher cognitive restraint eating behavior. Therefore, assessing weight loss history and eating behavior may be beneficial to better individualize obesity treatment.

## Introduction

Young adulthood is characterized by the transition from adolescence to adulthood, where different life changes, such as choice of education, occupation, residence, or partner, are essential parts of life [1]. Beyond this, neurocognitive maturity is expected to continue up to young adulthood in the mid-20s [2]. Therefore, weight management is challenging since central parts of the brain that involve, for example, decision-making, planning, regulation of emotions, and impulsivity, are under development [3]. In addition, the transition period is a phase where individuals are especially vulnerable to sociocultural ideals and social comparisons concerning their appearance [4].

Self-esteem is a construct that relates to negative or positive self-evaluation and feelings of self-worth [5]. Previously, it has been shown that children and adolescents with obesity report lower self-esteem than those with normal body weight in the same age range [6, 7]. Adolescents and young adults with obesity are at high risk for being bullied, teased, and discriminated [8]. Experiencing shame or social exclusion may contribute to impairment of self-esteem and feelings of internalized weight bias [9]. Low self-esteem and a poor body image in the context of experiences of weight stigma may contribute to mental health problems, dysfunctional eating behaviors, unhealthy weight loss methods, and future weight gain [8]. To repeatedly lose and regain weight have been associated with eating psychopathology in adults [10], and could be hypothesized to further increase psychological distress also among adolescents and young adults with obesity.

However, results regarding the association between self-esteem and obesity are mixed. For instance, when comparing Swedish adolescents aged 14–18 years with obesity to normal-weight peers, no difference in self-esteem was found [11]. Body dissatisfaction, however, was more prevalent among those with obesity. Further, a meta-analysis showed that pediatric obesity treatment may improve self-esteem and body image, while no association between change in self-esteem and weight outcomes was found [12]. Others, in turn, have suggested that weight loss induced by professionally led weight management programs alone may not improve self-esteem among adolescents with obesity [13].

Eating behaviors are central to weight management. It has been suggested that behaviors such as uncontrolled eating and emotional eating are associated with high body mass index (BMI, kg/m^2^) in the adult population [14]. Further, emotional eating has been associated with self-stigma and anxiety in female adolescents and with body shape in young male adolescents [15]. Cognitive restraint eating can be either beneficial or disadvantageous for self-regulation in weight management. Increased cognitive eating scores have, for example, been associated with enhanced weight loss outcomes among adults in intensive lifestyle intervention [16]. On the other hand, dietary restraint has been associated with increased BMI and unhealthy eating in women [17], contributing to more difficulties in weight management. In adolescents, low self-esteem and symptoms of depression have been associated with dysfunctional restrictive eating behavior [18]. Furthermore, it has been suggested that a high dietary restraint in children and adolescents may predict less successful weight loss outcomes of obesity treatment [19].

Weight loss results are often poor among children and adolescents with severe obesity when treated with lifestyle modification [20, 21]. Weight regain and loss to follow-up are common phenomena in behavioral interventions in this age group [20, 22]. Many young people may also experience a feeling of responsibility or pressure to try to lose weight on their own. These self-directed weight loss attempts may lead to repeated weight cycles instead of achieving sustained weight loss [23].

However, it is unclear how previous weight loss relates to self-esteem and eating behaviors in treatment-seeking adolescents and young adults with obesity at specialty clinics. Therefore, we aimed to assess the association between the amount and the frequency of previous weight loss with self-esteem and eating behavior in treatment-seeking young people in specialty obesity care. Based on previous research, we hypothesized that weight loss, or repeated weight loss, was associated with decreased self-esteem and increased uncontrolled eating, emotional eating, and cognitive restraint eating when compared to those with no previous weight loss events.

## Material and Methods

### Study Setting and Study Population

This cross-sectional study is part of a prospective open cohort study, *the Swedish Youth with Obesity (SYO)*, comprising adolescents and young adults aged 16–25 enrolled for obesity treatment at the Center for Obesity, Sweden. The center offers medical obesity treatment for young patients with a BMI ≥35 kg/m^2^, or BMI ≥30 kg/m^2^ with obesity-related conditions. Patients are referred to the Center for Obesity by physicians in primary health care, hospital-based specialty care, or school health services. Previous obesity treatment or weight loss attempts are not required for referral. Patients with a current or suspected eating disorder, alcohol use disorder, drug use, or severe mental health problems are not admitted to treatment. Instead, they are referred to other specialists.

Before treatment begins at the Center for Obesity, patients receive an invitation letter with written study information and a questionnaire including background data, weight loss history, eating behavior, self-esteem, and health- and lifestyle-related questions. The questionnaire is part of the clinical routine to assess patients' lifestyle and health ahead of treatment and is used in research if patients provide informed consent. Patients who do not understand the study information (e.g., intellectual disabilities) or do not provide written informed consent are not included in the cohort.

Inclusion criteria for this cross-sectional study were that participants attended their first enrollment visit at the Center for Obesity between May 5th, 2020 and June 2nd, 2022, signed informed consent, and answered the question about weight loss in the questionnaire. Of 282 potential participants, in total, 236 (83.7%) patients signed the informed consent during the study period. Twelve (5.1%) had not answered the weight loss question and were consequently excluded.

### Background Variables and Measures

We retrieved information on age at enrollment, sex, occupation, smoking status, and parental country of birth within a self-reported questionnaire. The parental country of birth was categorized into two groups: “Nordic country” if at least one parent was born in Denmark, Finland, Iceland, Norway, or Sweden, or “Other country” if both parents were born outside the Nordic countries. Smoking status was categorized as being a non-smoker or smoker. BMI (kg/m^2^) was calculated from measured weight (to the nearest 0.1 kg) and height (cm) collected at the enrollment visit.

### Weight Loss History

The amount of previous weight loss was assessed by the question, “Have you ever lost 5 kg or more in less than 1 year?.” If participants responded “yes,” they were also asked to specify the amount of weight loss; “Between 5 and 10 kg” or “More than 10 kg.” Further, they were asked about the frequency of such weight loss events, with response options ranging between 1 and 5 times or more. Women were asked to preclude weight loss associated with pregnancies. We categorized participants according to the amount of weight loss as follows: no weight loss, 5–10 kg, and >10 kg, and according to the frequency of weight loss to 0, 1, and ≥2 times. Participants with no weight loss, i.e., 0 times, became the reference group.

### Self-Esteem

Self-esteem was assessed by Rosenberg's Self-esteem Scale [5]. The self-esteem scale has been validated in high school students [5] and young adults [24]. It consists of five positive and five negative statements about one's beliefs about oneself, scored with a Likert scale from 0 to 3 (strongly agree, to strongly disagree). Positively worded items were reverse in the scoring. A score of <15 was used as an indicator of low self-esteem [25].

### Eating Behavior

The Three-Factor Eating Questionnaire-Revised21 (TFEQ-R21) was used to assess uncontrolled eating (9 items), emotional eating (6 items), and cognitive restraint eating (6 items) [26]. TFEQ-R21 has been validated in people with obesity [27]. Uncontrolled eating captures overeating due to hunger or exposure to external food cues [26]. Emotional eating, in turn, assesses the eating behavior responding to negative emotions. Finally, cognitive restraint eating captures a conscious and constant restriction of eating [26]. Scores were calculated for each domain, and each ranged from 0 to 100. A higher score indicates a greater degree of respective eating behavior.

### Statistical Analyses

We summarized participants' characteristics by using mean and standard deviations for continuous variables and study sample (n) and percentages (%) for categorical variables. We controlled variables for outliers and completeness. Missing data ranged from 0.5% (self-esteem), 0.9% (occupation), 1.3% (parental background), to 3.6% (smoking status). We checked the distribution (normality/skewness) for continuous variables, which were approximately normally distributed. One-way ANOVA was used to compare differences between groups in age, BMI, self-esteem score, and scores of uncontrolled eating, emotional eating, and cognitive restraint eating between the amount and the frequency of previous weight loss. χ^2^ tests were used to examine the distribution of sex, country of birth, occupation, smoking status, and self-esteem between the groups.

We fitted linear regression models to analyze the associations between the amount and the frequency of previous weight loss with self-esteem scores and scores of uncontrolled eating, emotional eating, and cognitive restraint eating, respectively. Besides unadjusted analyses, we also controlled for selected confounding variables based on subject matter knowledge, i.e., we adjusted for age, sex, BMI, and smoking status. Statistical significance was defined as a *p*-value <0.05. STATA 15.1 (Stata Corporation, Collage Station, TX, USA) was used to perform the statistical analyses.

## Results

Participant characteristics are presented in Table 1. Participants (*n* = 224) were primarily women, 72.8%. They were, on average, 21.0 years old and had a mean BMI of 41.8 kg/m^2^. Most of the participants had at least one parent born in a Nordic country (57.5%), were students (63.1%), and were non-smokers (78.7%). Participants reported a mean self-esteem score of 16.7, and more than half of them, 55.6%, reported a self-esteem score between 15 and 30. The mean scores for different eating behaviors were 46.0 for uncontrolled eating, 39.4 for emotional eating, and 45.9 for cognitive restraint eating.

The majority (71.0%) had previously lost ≥5 kg in a year. Almost one-third, 29.0%, had no previous weight loss, 40.6% had lost 5–10 kg, and 30.4% > 10 kg. In total, 27.2% reported having lost ≥5 kg once, and 43.8% had lost ≥5 kg twice or more. Participants who reported no previous weight loss were the youngest (mean age 20.0 years), compared to those who reported weight loss between 5 and 10 kg (mean age 20.8 years) and participants who reported >10 kg weight loss (mean age 22.2 years) (*p* = 0.0001). Participants with no weight loss had a mean BMI of 41.9 kg/m^2^, those who had lost between 5 and 10 kg had a mean BMI of 41.2 kg/m^2^, while those who had lost >10 kg had a slightly higher mean BMI of 42.7 kg/m^2^ (*p* = 0.37). On average, participants who reported having lost weight once were 21.0 years of age and those who had lost weight twice or more were older, 21.6 years (*p* = 0.002). Participants who reported never having lost weight and those who had lost twice or more had the same average BMI of 41.9 kg/m^2^. Those who had lost weight once had an average BMI at 41.6 kg/m^2^ (*p* = 0.94). Participants who reported no weight loss had an average self-esteem score of 17.0, shown in Table 2. We could not identify any statistically significant differences between the amount or the frequency of previous weight loss ≥5 kg with self-esteem. Further, we found no statistically significant differences in any eating behavior scores between participants in the different categories of the amount of weight loss or frequency of weight loss.

The results from linear regression models of associations between the amount and the frequency of previous weight loss, with self-esteem and eating behavior scores, are shown in Table 3. Participants who had lost between 5 and 10 kg had a statistically significantly higher score of 7.03 points (95% CI: 0.004–14.05) for cognitive restraint eating compared to those with no weight loss. Further, those who had lost ≥5 kg twice or more had a statistically significantly higher score of cognitive restraint eating by 8.32 points (95% CI: 1.20–15.43) compared to participants with no weight loss. No other statistically significant associations were seen.

## Conclusion

Among treatment-seeking adolescents and young adults with obesity, weight loss attempts were common. We found a statistically significant association between weight loss of 5–10 kg as well as between weight loss of ≥5 kg twice or more with cognitive restraint eating compared to those with no weight loss. However, we found no association between the amount or frequency of previous weight loss with self-esteem or other eating behaviors.

### Weight Loss History and Self-Esteem

Notably, 44.4% of participants in our study reported low self-esteem. Our participants had a mean BMI above 40 kg/m^2^. Obesity itself is shown to be associated with low self-esteem [28], which may explain why low self-esteem was so common. During adolescence, self-esteem seems to decline, especially in girls [29] and in individuals with obesity [30]. However, self-esteem is quite a stable construct within an individual. Those with low self-esteem at a young age tend to have low self-esteem even later on in life and vice versa [29]. Having experienced repeated weight loss and regain previously in life has been associated with low self-esteem among “dieters” in middle age [31]. In our study, participants who reported having lost >10 kg had the lowest mean self-esteem score. We also identified that participants who had lost >10 kg, were older and had a higher BMI than those with no weight loss or weight loss between 5 and 10 kg. Participants who reported more frequent weight loss were a bit older than those who had no weight loss, but still reported the same self-esteem score. In all, the mean self-esteem score in our study was lower than the score of 18.9 seen in another sample of Swedish adolescents with obesity [32]. This could perhaps be explained by the age difference among participants, 21.0 years in our study, compared to 16.5 years in the study by Järvholm et al. [32]. However, we did not find any differences between the amount or the frequency of previous weight loss ≥5 kg with self-esteem.

### Weight Loss History and Eating Behavior

Uncontrolled eating and emotional eating neither differed between the amount of previous weight loss nor the frequency of previous weight loss. We found that those who reported weight loss between 5 and 10 kg and weight loss of ≥5 kg twice or more, had higher cognitive restraint eating scores than those with no weight loss. Similar results were found in a Finnish adult population [33]. Those who had tried to lose weight three times or more had a significantly higher score for cognitive restraint eating, as well as a higher BMI than those who reported no weight loss attempts [33]. Others have also shown an association between high BMI and cognitive restraint eating behavior in young adults [34], children, and adolescents [35].

Although the results concerning the association between cognitive restraint eating and obesity are mixed, a cognitive restraint eating behavior seems necessary for weight loss and maintenance of weight loss. For instance, higher scores in cognitive restraint eating have been associated with lower weight in women with overweight or obesity [36]. However, it has also been suggested that a more cognitive restraint eating behavior may be a reaction to an increase in weight and, therefore, a surrogate marker for overeating and disinhibition [37]. This is opposite to the restraint theory which proposes that restraint eating behavior leads to overeating, which in turn contributes to obesity [38]. However, enhanced cognitive self-regulation for eating behaviors may be a pivotal factor in maintaining weight loss [37]. The associations between cognitive restraint eating with weight loss or repeated weight loss seen in the present study could also be explained by biological and genetic factors that promote obesity and weight regain by metabolic adaptation [39]. When individuals follow an energy-reduced diet and lose weight, there are significant changes in appetite and satiety-regulating hormones and peptides [40] that promote weight regain and contribute to difficulties in appetite regulation.

### Limitations and Strengths

The present study has several limitations and strengths. Because of the study's cross-sectional design, causal inferences of the direction of associations could not be assessed. Another limitation may have been that we used self-reported data and that information on weight loss history was collected retrospectively in categories: no weight loss, between 5 and 10 kg, and more than 10 kg, limiting the possibility to generate percentual weight loss. It can be challenging to remember the exact kilograms and the frequency of previous weight loss. Still, all participants had obesity and had actively sought treatment, thus it might be assumed they were likely to be aware of their weight loss history. Additionally, weight and height were objectively measured and used to calculate BMI at the baseline, which is a strength. Although data were self-reported, a strength of the present study was the use of Rosenberg's Self-esteem Scale [5, 24] and the TFEQ-R21 [26, 27], which both have shown high validity and reliability to assess self-esteem and eating behavior.

However, a shortcoming of all versions of TFEQ is that while indicating the degree of each respective eating behavior, they lack norms and clear cut-offs for what should be considered a low, moderate, or high degree of the behavior. Hence, in the clinical setting, the scores are only used as a basis for a dialogue where eating behaviors are assessed. Nevertheless, TFEQ has been widely used for subgroup analyses where, for example, women in the general population have been seen to report higher scores than men [41], indicating gender differences. Age may also play a role since older individuals score higher for cognitive restraint eating behavior, but lower for disinhibition [41]. Scores reported for emotional eating and uncontrolled eating in the present study are similar to how adolescents with a mean age of 16.5 years seeking bariatric surgery reported [32].

The small sample size may be a limitation. The confidence intervals from linear regression models were wide, which may have been due to the small number of participants in each weight loss category. Although statistically significant per definition, the lower level of the confidence interval for the association between a weight loss of 5–10 kg and cognitive restraint eating behavior was near zero (0.004), suggesting that we should interpret these results with caution.

Our study population was treatment-seeking adolescents and young adults with obesity from a real-world clinical setting. Therefore, it could be argued that they were a selected population and expected to have lost weight previously. Still, young people in late adolescence and young adulthood with obesity are seldomly studied. Therefore, there is a need to address adolescents' and young adults' health-related issues to a greater extent [42] and to improve the understanding of their specific needs in obesity treatment. It may limit the generalizability of our results that more than 70% of the treatment-seeking young individuals were women, even though obesity is nearly equally prevalent in women and men aged 16–29 in Sweden [43]. However, women are commonly more prone to seek treatment for their obesity, for example, approximately 80% of those who undergo bariatric surgery in Sweden are women [44]. Hence, our study population reflects the general young patients admitted to obesity treatment. An explorative study, such as focus groups or qualitative interviews, could complement these results for a deeper understanding of the lived experience of weight loss, eating behaviors, and self-esteem in adolescents and young adults with obesity.

In conclusion, weight loss attempts were common among adolescents and young adults with obesity before treatment at the specialty obesity clinic. Previous weight loss was not associated with self-esteem, uncontrolled eating, or emotional eating. However, both the amount and the frequency of previous weight loss were associated with cognitive restraint eating behavior. Therefore, a careful assessment of weight loss history and eating behavior may be a pathway to identify possible unhealthy and rigid eating behaviors in adolescents and young adults with obesity, to better support them in their weight management.

## Statement of Ethics

This study was performed in line with the principles of the World Medical Association Declaration of Helsinki.

## Conflict of Interest Statement

The authors have no conflicts of interest to declare.

## Funding Sources

Author Liisa Tolvanen was supported by the Research School in Family medicine and primary care organized by Karolinska Institutet and Region Stockholm. Author Stephanie E. Bonn obtained funding from SFO-V, Karolinska Institutet. Author Ylva Trolle Lagerros was funded by Region Stockholm clinical research appointment (Grant number DNR RS 2019-1140). The funders had no role in the preparation of data or the manuscript.

## Author Contributions

Liisa Tolvanen and Ylva Trolle Lagerros designed the study. Anne Christenson and Ylva Trolle Lagerros were overall responsible for the data collection. Liisa Tolvanen and Helén Eke conducted the statistical analysis. Stephanie E. Bonn and Ylva Trolle Lagerros supervised the analyses. The interpretation was made in collaboration with all authors. Liisa Tolvanen wrote the first draft of the manuscript. All authors critically reviewed the manuscript and agreed on the final version.

## Data Availability Statement

All data generated and analyzed during this study are included in this article. Further inquiries can be directed to the corresponding author.

## Figures and Tables

**Table 1:** Participants’ characteristics (*n* = 224)

	Mean	SD
Age, years	21.0	3.0
Weight, kg	121.0	24.2
Height, cm	169.9	10.7
BMI, kg/m^2^	41.8	6.6
Self-esteem score^a^	16.7	8.0
Eating behavior scores^b^		
Uncontrolled eating	46.0	24.4
Emotional eating	39.4	29.2
Cognitive restraint eating	45.9	21.0

	*n*	%
Self-esteem cut-off scores^a^		
<15	99	44.4
15–30	124	55.6
Sex		
Women	163	72.8
Men	59	26.3
Other	2	0.9
Parental country of birth^c^		
Nordic country	127	57.5
Other country	94	42.5
Occupation		
Student	140	63.1
Employed	56	25.2
Other^d^	26	11.7
Smoking status		
Non-smoker	170	78.7
Smoker	46	21.3

SD, standard deviation.

a Rosenberg's Self-esteem Scale scores.

b TFEQ-R21 scores.

c At least one parent born in a Nordic country; Both parents born outside the Nordic countries.

d E.g., parental leave, sick leave, unemployed.

**Table 2:** Differences in self-esteem and eating behavior between the amount of previous weight loss and the frequency of previous weight loss of ≥5 kg (*n* = 224)

	Reference category	Amount of weight loss			Frequency of weight loss of ≥5 kg		
	no weight loss (*n* = 65)	5–10 kg (*n* = 91)	>10 kg (*n* = 68)	*p* value^a^	once (*n* = 61)	twice or more (*n* = 98)	*p* value^a^
	*n* (%)	*n* (%)	*n* (%)		*n* (%)	*n* (%)	
Self-esteem cut-off^b^							
<15	27 (41.5)	39 (42.9)	33 (49.2)	0.62	32 (52.5)	40 (41.2)	0.33
15–30	38 (58.5)	52 (57.1)	34 (50.8)		29 (47.5)	57 (58.8)	

	mean (SD)	mean (SD)	mean (SD)	*p* value^c^	mean (SD)	mean (SD)	*p* value^c^
Self-esteem^b^	17.0 (8.5)	17.6 (8.4)	15.1 (7.0)	0.16	15.7 (8.4)	17.0 (7.6)	0.55
Eating behavior^d^							
Uncontrolled eating	43.1 (27.4)	45.2 (20.8)	49.9 (25.8)	0.26	47.1 (21.1)	47.3 (24.4)	0.53
Emotional eating	37.4 (30.5)	37.2 (28.6)	44.2 (28.4)	0.26	42.4 (27.6)	38.9 (29.4)	0.62
Cognitive restraint eating	41.7 (20.0)	48.8 (20.7)	46.1 (21.9)	0.12	45.3 (22.1)	49.1 (20.5)	0.09

SD, standard deviation.

a χ^2^ test.

b Rosenberg's Self-esteem Scale scores.

c One-way ANOVA.

d TFEQ-R21 scores.

**Table 3:** Results from linear regression models between self-esteem score, eating behavior scores with the amount of previous weight loss, and the frequency of previous weight loss of ≥5 kg

	Amount of weight loss	Model 1^a^		Model 2^b^	
		β-coefficient	95% CI	β-coefficient	95% CI
Self-esteem^c^					
Self-esteem score	No weight loss	Reference		Reference	
	5–10 kg	0.52	−2.04 to 3.09	−0.77	−3.32 to 1.79
	>10 kg	−1.89	−4.64 to 0.87	−1.94	−4.75 to 0.87
Eating behavior^d^					
Uncontrolled eating	No weight loss	Reference		Reference	
	5–10 kg	2.11	−5.73 to 9.96	2.78	−5.42 to 10.99
	>10 kg	6.79	−1.59 to 15.16	7.35	−1.66 to 16.35
Emotional eating	No weight loss	Reference		Reference	
	5–10 kg	−0.20	−9.57 to 9.17	0.28	−9.50 to 10.07
	>10 kg	6.82	−3.18 to 16.82	6.00	−4.76 to 16.73
Cognitive restraint eating	No weight loss	Reference		Reference	
	5–10 kg	7.11	0.40 to 13.81	7.03	0.004 to 14.05
	>10 kg	4.37	−2.79 to 11.53	4.51	−3.20 to 12.22

	Frequency of weight loss of ≥5 kg	Model 1^a^		Model 2^b^	
		β-coefficient	95% CI	β-coefficient	95% CI
Self-esteem^c^					
Self-esteem score	No weight loss	Reference		Reference	
	Once	−1.32	−4.15 to 1.51	−1.86	−4.63 to 0.90
	Twice or more	0.02	−2.53 to 2.57	−0.74	−3.34 to 1.86
Eating behavior^d^					
Uncontrolled eating	No weight loss	Reference		Reference	
	Once	4.02	−4.61 to 12.66	3.83	−5.08 to 12.73
	Twice or more	4.17	−3.59 to 11.92	5.13	−3.24 to 13.50
Emotional eating	No weight loss	Reference		Reference	
	Once	4.99	−5.32 to 15.31	3.81	−6.81 to 14.43
	Twice or more	1.44	−7.83 to 10.70	1.53	−8.45 to 11.52
Cognitive restraint eating	No weight loss	Reference		Reference	
	Once	3.61	−3.74 to 10.95	3.02	−4.55 to 10.59
	Twice or more	7.38	0.79 to 13.98	8.32	1.20 to 15.43

a Crude model.

b Adjusted for age, sex, BMI, and smoking status.

c Rosenberg's Self-esteem Scale scores.

d TFEQ-R21 scores.
